# Complete Genome Sequence of an Enterotoxigenic *Bacteroides fragilis* Clinical Isolate

**DOI:** 10.1128/genomeA.00450-15

**Published:** 2015-05-07

**Authors:** Anastasia S. Nikitina, Daria D. Kharlampieva, Vladislav V. Babenko, Dmitriy A. Shirokov, Maria T. Vakhitova, Aleksandr I. Manolov, Andrei N. Shkoporov, Anastasia E. Taraskina, Valentin A. Manuvera, Vassili N. Lazarev, Elena S. Kostryukova

**Affiliations:** a Scientific Research Institute of Physical-Chemical Medicine, Moscow, Russia; b Moscow Institute of Physics and Technology, Dolgoprudny, Moscow Region, Russia; c Pirogov Russian National Research Medical University, Moscow, Russia; d I.P. Pavlov First Saint Petersburg State Medical University, Saint Petersburg, Russia; e Kashkin Research Institute of Medical Mycology, North-Western State Medical University named after I.I. Mechnikov, Saint Petersburg, Russia

## Abstract

Here we present the complete genome sequence of *Bacteroides fragilis* isolate BOB25. It is an enterotoxigenic isolate that was obtained from a stool sample of a patient with dysbiosis.

## GENOME ANNOUNCEMENT

*Bacteroides fragilis* is a Gram-negative, obligate anaerobic, rod-shaped bacterium that comprises about 1 to 2% of the normal human colonic microflora and is critical to systematic and mucosal immunity and host nutrition (1). However, it is also an opportunistic pathogen, being the leading anaerobic isolate in clinical specimens, bloodstream infections, and abdominal abscesses (2). Pathogenic strains termed enterotoxigenic *B. fragilis* (ETBF) are implicated in inflammatory diarrhea and have been associated with colorectal cancer in humans (3, 4). The *B. fragilis* toxin (BFT), a 20-kDa, secreted, zinc-dependent metalloprotease, has been identified as a specific virulence factor for ETBF, although the mechanisms of the intracellular processing, secretion, and those accounting for the variation of expression have not yet been elucidated (5).

The genome of enterotoxigenic *B. fragilis* was sequenced by two sequencing methods. One fragment library was sequenced using a GS FLX+ genome sequencer (Roche 454 Life Science, USA) with 13-fold coverage and median length of 812 bp. Another library was sequenced with the PGM sequencer system (Applied Biosystems, USA) with 16-fold coverage and median length of 206 bp. The raw reads from both runs were then combined and filtered using the spectral alignment error correction tool SAET 3 (6) and yielded 685,605 reads with overall coverage of 29-fold. Primary assembly was conducted with Newbler version 2.9, resulting in 42 contigs (>1,000 bp in size) and an estimated genome size of 5.6 Mb. In order to fill the gaps between contigs, corresponding amplicons were generated and sequenced using an ABI Prism Genetic Analyzer 3730XL following the manufacturer’s instructions (Applied Biosystems).

The complete genome of *B. fragilis* isolate BOB25 has a length of 5,282,232 bp and a G+C content of 43.2% The annotation was performed using the NCBI Prokaryotic Genome Annotation Pipeline (http://www.ncbi.nlm.nih.gov/genome/annotation_prok) and identified a total of 4,112 coding sequences (CDSs), 18 rRNA genes, and 70 tRNA genes. The most important difference between the ETBF strain and published genome sequences of nontoxigenic (NTBF) strains (NCTC9343, YCH46, and 638R [7–9]), which is also responsible for the pathogenic behavior of this strain, is the presence of a *B. fragilis* pathogenicity island (BfPAI) located within the conjugative transposon CTn86 of 63,282 bp. Interestingly, two copies of this transposon are present in the genome of BOB25, separated by 934,125 bp and oriented in opposite directions. Similar to those previously characterized (10), the pathogenicity island of this isolate contains the enterotoxin gene *bft-2* and the second metalloprotease gene (*mpII*), and it is flanked by putative mobilization genes (*bfmA*, *bfmB*, and *bfmC*). In addition, there are several regions that show no similarity to NTBF genomic sequences, most of which are mobile elements like prophages and conjugative transposons. However, some of these regions appear to be involved in capsular polysaccharide synthesis, making them attractive targets for further research. Finally, unlike most of the nontoxigenic strains, the complete genome sequence of the ETBF isolate BOB25 does not contain any plasmids.

### Nucleotide sequence accession numbers. 

The genome sequence of *B. fragilis* isolate BOB25 was deposited at GenBank under the accession number CP011073. The version described in this paper is the first version, CP011073.1.
